# Epigenetic mechanisms linking the perinatal environment, the placenta, and maternal and child health outcomes: evidence for sexual dimorphism

**DOI:** 10.1093/eep/dvag012

**Published:** 2026-04-02

**Authors:** Hadley J Hartwell, Rebecca C Fry

**Affiliations:** Department of Environmental Sciences and Engineering, Gillings School of Global Public Health, University of North Carolina, Chapel Hill, NC 27599, United States; Department of Environmental Sciences and Engineering, Gillings School of Global Public Health, University of North Carolina, Chapel Hill, NC 27599, United States

**Keywords:** epigenetics, Developmental Origins of Health and Disease (DOHaD), placenta, maternal-child health, environmental exposures

## Abstract

Exposure to adverse environments early in life can shape health trajectories across the lifespan. A key mechanism by which this life-long reprogramming occurs is via epigenetic modifications, including altered DNA methylation (DNAm), histone modifications, and microRNA (miRNA) regulation. This invited perspective highlights key human population studies and selected animal studies from our group and collaborators that have examined toxicant exposure occurring during or prior to pregnancy including metals, pharmaceuticals, microorganisms, air pollution, and socioeconomic stressors and their impact on the epigenome. Exposure to these substances is associated with altered epigenetic patterning in fetal blood and placenta, often in a gene- and sex-specific manner. This gene specificity may be tied to the transcription factor occupancy, where environmental exposures alter transcription factor binding at regulatory regions, influencing downstream epigenetic patterns. In relation to adverse health outcomes, these epigenetic modifications have been associated with adverse pregnancy outcomes such as preeclampsia as well as neonatal health (i.e. preterm birth, retinopathy of prematurity, chronic lung disease, and congenital heart defects). Additionally, these epigenetic alterations have been associated with outcomes later in childhood, including cognition, neurodevelopmental disorders [e.g. autism spectrum disorder (ASD) and attention-deficit/hyperactivity disorder (ADHD)], obesity, metabolic dysregulation, asthma, and immune dysfunction. Collectively, these studies highlight the relationships among early-life environmental factors, epigenetic biomarkers, and maternal and child health outcomes.

## Graphical Abstract

**Graphical Abstract:**
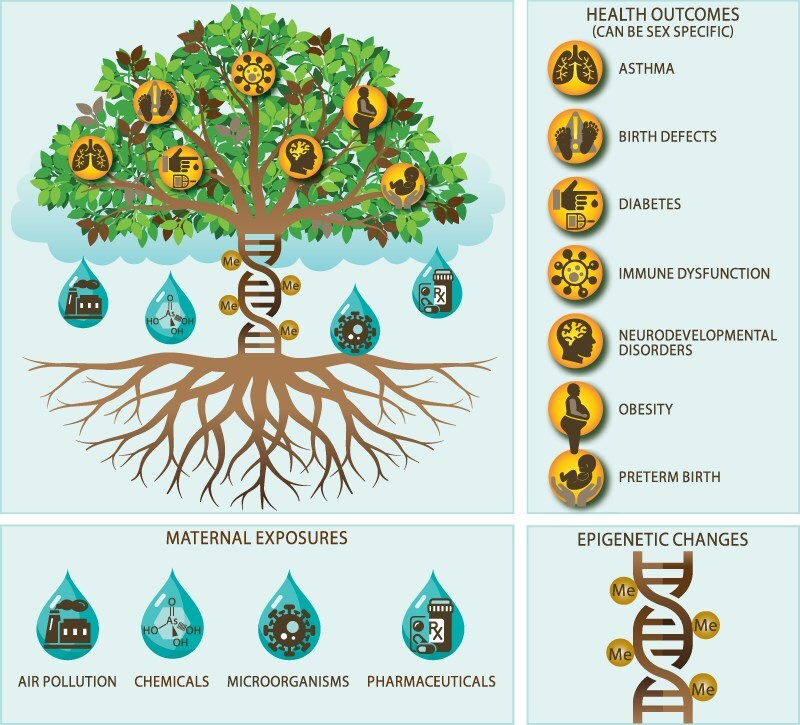

## Introduction

**Figure 1:**
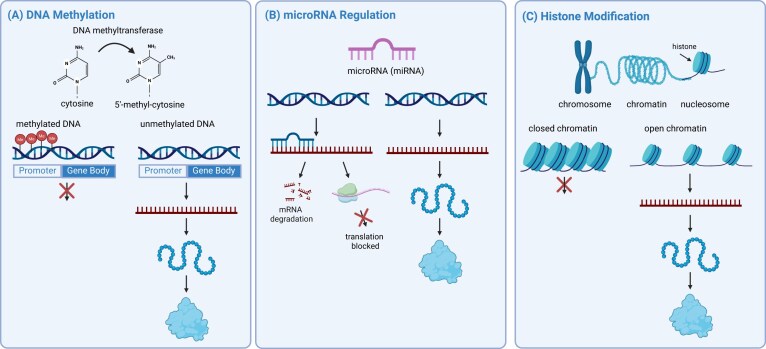
Mechanisms of epigenetic regulation of gene expression. Diagram illustrating three key epigenetic mechanisms: (A) DNA methylation, where methylation of cytosine residues by DNA methyltransferases may lead to reduced gene expression; (B) microRNA (miRNA) regulation, in which miRNA binding to complementary mRNA triggers mRNA degradation or represses mRNA translation; and (C) histone modification, where chromatin structure influences transcriptional activity with closed chromatin repressing gene expression, while open chromatin permitting transcription.

**Figure 2:**
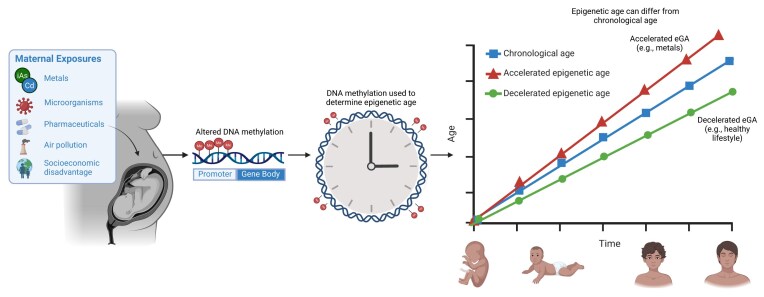
Epigenetic clock. Conceptual illustration of how maternal environmental exposures influence biological aging. Epigenetic clocks estimate biological age from DNA methylation patterns at specific CpG sites. These sites gain or lose methylation in predictable ways across the lifespan, enabling models to predict biological age independently of chronological age. Divergence between predicted and actual age reflects epigenetic age acceleration or deceleration.

According to the Developmental Origins of Health and Disease (DOHaD) hypothesis, early-life exposures play important roles in shaping health across the lifespan [1]. Environmental chemicals and other stressors have been associated with epigenetic changes that influence gene regulation without altering the underlying DNA sequence [2]. The proposed primary epigenetic mechanisms include DNA methylation (DNAm), microRNA (miRNA) regulation, and histone modifications (Fig. 1). Alterations in DNAm can influence gene transcription, leading to increased or decreased gene expression [3]. Non-coding RNAs, including miRNAs and long non-coding RNAs, regulate gene expression through multiple mechanisms, such as modulating chromatin accessibility and influencing the stability or translation of target messenger RNAs [4]. Histone post-translational modifications impact gene expression in a variety of different ways including influencing chromatin structure. In addition, specific combinations of histone marks can poise genes for activation or repression depending on developmental or environmental cues [5]. Notably, altered DNAm at specific cytosines have been shown to be associated with biological aging and analyzed as epigenetic clocks (Fig. 2) [6]. This perspective includes a summary of studies that have assessed epigenetic alterations, focusing primarily on changes in DNAm and miRNA expression as key mechanisms linking environmental exposures to gene regulation.

A strong body of literature, including work from our lab and collaborators, highlights associations among exposure to a range of environmental factors including metals, microorganisms, pharmaceuticals, socioeconomic stressors, and air pollution with epigenetic modifications [7–16]. These epigenetic changes have been associated with adverse pregnancy, neonatal, and childhood outcomes. This invited perspective highlights findings from our laboratory and collaborators that detail these findings in relation to health effects across these three life stages. First, we highlight studies that have focused on pregnancy outcomes, with an emphasis on preeclampsia. Second, we explore neonatal health, highlighting epigenetic mechanisms underlying preterm birth (PTB), retinopathy of prematurity (ROP), chronic lung disease (CLD), and Tetralogy of Fallot (TOF). Third, we highlight the studies that examine child health outcomes including cognition, neurodevelopmental disorders, obesity, asthma, and immune dysregulation. Together, these findings illustrate the central role of the epigenome, and in many cases the placenta specifically, in linking early-life exposures to lifelong health trajectories.

Building on this foundation, research from our group and others has advanced the field of environmental epigenetics by demonstrating that maternal exposures during pregnancy leave measurable and biologically meaningful imprints on the placental and fetal epigenome. Through cohort studies, including the Biomarkers of Exposure to Arsenic (BEAR), Extremely Low Gestational Age Newborns (ELGAN), Environmental influences on Child Health Outcomes (ECHO), and the New Hampshire Birth Cohort, we and our collaborators have shown that DNAm and miRNA signatures in the placenta and neonatal blood are associated with outcomes ranging from preeclampsia and PTB to neurodevelopmental disorders, obesity, and asthma. Our studies have further revealed that these epigenetic signatures are often sex-specific, highlighting fundamental differences in developmental susceptibility between males and females. Importantly, these findings have shifted the field beyond simple associations, positioning the placenta as a central mediator of exposure-related risk and identifying reproducible epigenetic biomarkers with future translational potential for risk prediction and early intervention. While this invited perspective draws heavily on insights from our laboratory, these findings align with results from multi-cohort studies, collectively underscoring the translational potential of epigenetic biomarkers to predict long-term health trajectories [17–19].

## Pregnancy outcomes

Healthy pregnancies depend on many factors, chief among them is the proper formation and function of the placenta [20]. Placental health can be disrupted by molecular changes to the genome and epigenome, which in turn alter placental function. A wide range of adverse exposures, including metals, pharmaceuticals, microorganisms, and socioeconomic adversity, have been shown to alter the placental epigenome [12, 14–16, 21–24]. Because of this sensitivity, epigenomic changes are increasingly recognized as informative biomarkers of prenatal exposures. Recent analyses of placental tissues from the ELGAN cohort suggest that miRNA expression and DNAm may serve as more reliable indicators of perinatal health than transcriptomic data [25]. Importantly, many of these epigenetic modifications display sex-specific differences, highlighting the placenta’s distinct responses to environmental cues depending on fetal sex [16, 23, 26, 27]. Shown in Table 1 is list of selected studies examining the relationship between epigenetic changes and pregnancy outcomes.

**Table 1:** Epigenetic studies focusing on pregnancy-related health outcomes. ^a^

Study	Exposure or condition	Biological sample	Epigenetic changes	Health or developmental outcome	Sex-specific	Cohort
Clark *et al*. [25]; PMCID: 8478332	Multi-omics predictors	Placental tissue	Altered DNAm and miRNA	Predictors of perinatal outcomes: placental cell growth, blood vessel development, and fetal weight		ELGAN
Huff *et al*. [15]; PMCID: 11854159	Copper and manganese	Umbilical cord and placental tissue	Altered epigenetic clock	Reduced placental epigenetic aging in males	Yes	ELGAN
Bulka *et al*. [22]; PMID: 38281 400	Inorganic arsenic and cadmium	Umbilical cord and placental tissue	Altered DNAm	Potential effects on placental nutrient and chemical transport		ELGAN
Sanders *et al*. [24]; PMCID: 3962531	Cadmium	Maternal and newborn leukocytes	Altered DNAm	Demonstrated exposure-associated DNAm alterations in mother–baby pairs, supporting Cd as a prenatal epigenetic disruptor		CEHI
Addo *et al*. [14]; PMCID: 6682751	Acetaminophen	Placental tissue	Altered DNAm	Placental blood flow, fetal growth		ELGAN
Tomlinson *et al*. [12]; PMCID: 5730116	Placental microbiome	Placental tissue	Altered DNAm	Altered immune and inflammation pathways (NF-κB pathway)		ELGAN
Santos *et al*. [16]; PMCID: 6615526	Socioeconomic adversity	Placental tissue	Altered DNAm	Altered DNAm in immune and stress-response genes	Yes	ELGAN
Clark *et al*. [23]; PMCID: 8333418	Pre-pregnancy body mass index	Placental tissue	Altered miRNA	Impaired fetal growth and metabolism	Yes	ELGAN
Brooks *et al*. [11]; PMCID: 5156314	Cadmium	Placental tissue	Altered miRNA	Placental angiogenesis disruption and preeclampsia risk		UNC Hospitals
Martin *et al*. [28]; PMCID: 4624949	Preeclampsia	Placental tissue	Altered DNAm	Biomarkers for preeclampsia: methylation of *TGF-β* genes		UNC Hospitals
Ladd-Acosta *et al*. [27]; PMCID: 9958528	Preeclampsia and gestational diabetes	Umbilical cord blood	Altered epigenetic clock	Decelerated epigenetic gestational age, especially in females	Yes	ECHO
Eaves *et al*. [26]; PMCID: 7607407	Sex-specific miRNA regulation	Placental tissue	Altered miRNA	Sex-based variation in placental function	Yes	ELGAN

a Summary of selected studies linking maternal exposures to pregnancy health outcomes through epigenetic mechanisms. Columns indicate the exposure or condition assessed, biological sample analyzed, observed epigenetic change, associated health or developmental outcome, evidence of sex-specific effects, and the study cohort. Epigenomic alterations include altered DNA methylation (DNAm), microRNA (miRNA), and epigenetic clocks. NF-κB: nuclear factor kappa B; TGF-β: transforming growth factor beta; ELGAN: Extremely Low Gestational Age Newborn cohort; CEHI: Children’s Environmental Health Initiative; ECHO: Environmental influences on Child Health Outcomes cohort; UNC Hospitals: University of North Carolina Hospitals.

Among environmental exposures, metals are well-established modulators of the placental and neonatal blood epigenome. Prenatal exposure to metals such as iAs, Cu, and Mn is associated with altered DNAm in key placental genes, slower placental epigenetic aging, and altered placental cell composition, suggesting potential molecular pathways through which early metal exposure may influence long-term health [15, 22, 24]. In the ELGAN cohort, Cu and Mn levels in umbilical cord tissues were associated with reduced placental epigenetic age, specifically in males [15]. In another ELGAN study, analysis of placental and cord tissues highlighted the association between iAs, Cd, and altered DNAm in genes involved in placental nutrient and chemical transport [22]. These findings are consistent with findings from Sanders *et al*. showing that maternal Cd exposure is associated with widespread DNAm changes in maternal and newborn leukocytes [24]. Pharmaceutical exposures during pregnancy can also leave distinct epigenetic signatures in the placenta, with potential functional consequences for placental physiology. For example, acetaminophen use has been associated with DNAm changes in genes that are involved in maintaining placental blood flow and fetal growth [14]. It should be noted that while acetaminophen use during pregnancy has been associated with DNAm changes, these studies did not examine adverse fetal health outcomes. Beyond chemical exposures, biological agents also shape the placental epigenome. Bacterial measures in the placenta have been linked to widespread DNAm changes in immune and inflammatory pathway genes [12]. Evidence from 84 ELGAN placental samples further showed that the placental microbiome altered DNAm related to nuclear factor kappa B (NF-κB) signaling [12]. Future research should investigate whether there are mechanistic links between the placental microbiome and adverse pregnancy outcomes.

In addition to chemical and microbial exposures, non-chemical stressors play a role in influencing the placental epigenome. Prenatal maternal socioeconomic adversity has been linked to differential DNAm at multiple CpG sites [16]. Specifically, in the ELGAN cohort, analysis of placentas demonstrated that adversity was associated with DNAm changes in immune and stress-response genes. Maternal health before and during pregnancy likewise influences the placental epigenome, with potential lifelong implications for offspring. For example, in the ELGAN cohort, low pre-pregnancy body mass index (BMI) was associated with widespread alterations in placental miRNA expression [23]. The mRNA targets of these miRNAs were enriched for their roles in pathways related to nutrient metabolism and angiogenesis, suggesting that maternal underweight status may affect fetal growth and development through epigenetically mediated mechanisms.

Epigenetic alterations in the placenta have emerged as important contributors to the pathophysiology of preeclampsia, a pregnancy complication characterized by high blood pressure [29]. Preeclamptic placentas display disrupted expression of miRNAs within the transforming growth factor beta (TGF-β) pathway, a key regulator of placental vascular development [11,28]. In a cohort from UNC Hospitals, Cd exposure was linked to altered miRNA expression influencing preeclampsia risk [11]. In the same cohort, altered DNAm of *TGF-β* pathway genes was identified as a potential biomarker of the condition [28]. Evidence from umbilical cord blood samples in the ECHO cohort further demonstrated that preeclampsia and gestational diabetes were associated with decelerated epigenetic gestational age (eGA) in newborns [27]. Together, these findings suggest that placental epigenetic dysregulation, particularly within angiogenesis-related pathways, may underlie both the development of preeclampsia and its downstream consequences for child health.

Underlying these placental epigenetic modifications is a sex-specific theme [15, 16, 23, 26, 27]. These studies have identified thousands of sex-specific differentially methylated CpG sites in the placenta, involving genes related to immune function, growth signaling, and membrane transport. Specifically, analysis of placental tissues identified sex-specific miRNA regulation of placental function [26]. These findings offer important insights into how placental epigenetic differences may contribute to sex-based variations in infant outcomes and responses to prenatal environments. These findings reinforce the role of fetal sex as a critical modifier of placental molecular responses to environmental exposures.

Collectively, these studies demonstrate that placental epigenetic profiles capture a diverse array of environmental, biological, and social exposures, often in a sex-specific manner. This growing body of evidence underscores the placenta’s central role as both a target and a mediator of *in utero* influences on lifelong health.

## Neonatal health outcomes

Neonatal health represents a pivotal stage in early development, influencing survival and shaping long-term physical, cognitive, and metabolic trajectories. Complications during this period can have enduring effects across the lifespan [1]. Emerging evidence indicates that maternal environmental exposures during pregnancy can affect neonatal outcomes through epigenetic mechanisms, particularly changes in DNAm and miRNA expression. These molecular signatures provide insight into developmental processes and may serve as early biomarkers of risk for predicting neonatal complications such as preterm PTB, ROP, and CLD [30–34]. In addition, epigenetic disruptions linked to these exposures have been observed in congenital conditions such as TOF, a structural heart defect [13]. Table 2 summarizes studies from our group and collaborators highlighting the association between epigenetic changes and neonatal outcomes.

**Table 2:** Epigenetic studies focusing on neonatal health outcomes.^a^

Study	Exposure or condition	Biological sample	Epigenetic changes	Health or developmental outcome	Sex-specific	Cohort
Vidal *et al*. [10]; PMCID: 4502530	Cadmium, iron, and zinc	Umbilical cord blood	Altered DNAm	Lower birthweight	Yes	NEST
Rojas *et al*. [8]; PMCID: 4274382	Inorganic arsenic	Umbilical cord blood	Altered DNAm	Altered gestational age and head circumference		BEAR
Rager *et al*. [9]; PMCID: 4023469	Inorganic arsenic	Umbilical cord blood	Altered miRNA	Cancer, diabetes, and immune-related pathways		BEAR
Manuck *et al*. [30]; PMCID: 6002888	Recurrent PTB + progesterone	Placental tissue	Altered DNAm	Downregulated nitric oxide genes		UNC PTB Biobank
Manuck *et al*. [31]; PMCID: 8173522	PTB	Maternal blood	Altered miRNA	Preterm birth prediction using nitric oxide pathway-related miRNA expression		UNC PTB Biobank
Gascoigne *et al*. [32]; PMCID: 10920398	Maternal biological age	Maternal blood	Altered epigenetic clock	Risk of PTB linked with accelerated epigenetic age		UNC PTB Biobank
Eaves *et al*. [35]; PMCID: 10289042	Systemic inflammation	Placental tissue and neonatal blood	Altered DNAm	Inflammatory outcomes in preterm infants		ELGAN
Bulka *et al*. [33]; PMCID: 6607927	Retinopathy of prematurity	Placental tissue	Altered DNAm	Altered DNAm of angiogenesis and inflammation genes linked to ROP risk		ELGAN
Santos *et al*. [36]; PMCID: 10464100	Sex-specific DNA methylation	Placental tissue and neonatal blood	Altered DNAm	Sex differences in epigenetic aging	Yes	ELGAN
Jackson *et al*. [34]; PMCID: 9160210	Chronic lung disease	Placental tissue	Altered DNAm	Biomarkers of CLD in preterm infants	Yes	ELGAN
Luben *et al*. [13]; PMID: 40269475	Air pollution	Newborn dried blood spots	Altered DNAm	Epigenetic changes in genes related to metabolism, inflammation, immune function, and Tetralogy of Fallot risk		NC Birth Defects
Bulka *et al*. [37]; PMCID: 9980626	Gestational age and sex	Placental tissue	Altered DNAm	Sex-specific placental CpG sites related to gestational age	Yes	ECHO
Hartwell *et al*. [38]; PMCID: 11846692	Inorganic arsenic	Mouse liver	Altered DNAm	Altered imprinted and diabetogenic genes	Yes	Preconception Mouse Model
Shang *et al*. [39]; PMID: 40504410	Inorganic arsenic	Mouse liver	Altered DNAm	Altered imprinted and diabetogenic genes	Yes	Preconception Mouse Model

a Summary of selected studies linking maternal exposures during pregnancy to neonatal health outcomes through epigenetic mechanisms. Columns indicate the exposure or condition assessed, biological sample analyzed, observed epigenetic change, associated health or developmental outcome, evidence of sex-specific effects, and the study cohort. Epigenomic alterations include altered DNA methylation (DNAm), microRNA (miRNA), and epigenetic clocks. PTB: pre-term birth; CLD: chronic lung disease; ROP: retinopathy of prematurity; NEST: Newborn Epigenetics Study; BEAR: Biomarkers of Exposure to Arsenic; UNC PTB Biobank: University of North Carolina Pre-term Birth Biobank; ELGAN: Extremely Low Gestational Age Newborn cohort; ECHO: Environmental influences on Child Health Outcomes cohort.

Maternal exposure to toxic metals is among the most widely studied environmental factors influencing neonatal health through the epigenome. In the Newborn Epigenetics Study (NEST) cohort, analysis of umbilical cord blood samples showed that elevated maternal Cd levels during early pregnancy were associated with lower birth weight as well as sex-specific DNAm alterations in imprinted genes in offspring [10]. Low maternal iron (Fe) status may exacerbate these effects, while essential metals such as zinc (Zn) appear to mitigate them. Evidence from the BEAR pregnancy cohort in Gómez Palacio, Mexico, further illustrates these relationships. In this cohort, prenatal iAs exposure in mother–infant pairs was associated with widespread DNAm changes in umbilical cord blood, including at loci involved in transcription factor (TF) binding, which correlated with shorter gestational age and smaller head circumference [8]. In a related analysis of mother–infant pairs from the same cohort, iAs exposure was also linked to increased expression of 12 miRNAs predicted to regulate genes in cancer, diabetes, and immune-related pathways [9]. Collectively, these findings highlight the complex and multifaceted ways maternal toxic metal exposure can shape the neonatal epigenome and, in turn, fetal growth and development.

PTB is one of the most consequential neonatal outcomes, contributing substantially to both morbidity and mortality worldwide [40]. Epigenetic markers have emerged as promising predictors of PTB risk, including altered DNAm patterns in the placenta and changes in maternal blood miRNA expression, particularly in pathways related to nitric oxide signaling [30, 31]. In the UNC PTB Biobank, analysis of placentas demonstrated that recurrent PTB is associated with altered DNAm leading to downregulation of nitric oxide-related genes [30]. In the same cohort, analysis of maternal blood samples showed that nitric oxide pathway-related miRNA expression could be used to predict PTB risk [31]. Beyond these pathway-specific signatures, analysis of maternal blood samples from the UNC PTB Biobank revealed that maternal biological aging, measured using AgeAccelGrim DNAm clock, was also linked to PTB, with accelerated aging significantly increasing the likelihood of preterm delivery [32].

Early-life inflammation represents a critical pathway through which epigenetic mechanisms shape neonatal health. In the ELGAN cohort, placental tissues and neonatal blood samples were analyzed to investigate this relationship [35]. Results showed that intermittent or sustained systemic inflammation is associated with DNAm changes in the placenta, whereas acute, day-one inflammation corresponds to methylation alterations in neonatal blood. These findings suggest that inflammatory exposures during key developmental windows elicit tissue-specific epigenetic responses, which may contribute to differential regulation of immune function and developmental trajectories in neonates.

These inflammatory processes are particularly relevant to ROP, a disorder marked by abnormal retinal vascular development and is strongly associated with inflammatory and immune dysregulation [41, 42]. In a recent study of ELGAN placental samples, DNAm at CpG sites within genes involved in inflammation, angiogenesis, and neurotrophic signaling was associated with ROP in extremely preterm infants [33]. These findings underscore the potential of epigenetic biomarkers to enable earlier predictions of ROP risk and to inform interventions aimed at reducing disease severity.

Sex-specific patterns in neonatal epigenetic regulation further refine our understanding of susceptibility to adverse outcomes. In the ELGAN cohort, placentas derived from males exhibit hypermethylation enriched for keratinocyte differentiation pathways, while females show distinct hypermethylation signatures in neonatal blood [36]. Additional examination of ELGAN placental samples identified DNAm changes at 49 sites within genes critical for fetal lung development, serving as potential biomarkers of CLD risk [34]. Similarly, analyses of ECHO cohort placentas from female and male neonates demonstrated sex-specific associations between gestational age and placental DNAm [37]. These studies underscore the importance of considering both sex-specific epigenetic patterning when assessing neonatal risk profiles.

Interestingly, epigenetic changes have been associated with congenital structural outcomes, including TOF, a complex cardiac defect. In the NC Birth Defects cohort, gestational exposure to air pollution, assessed across newborn dried blood spots, was associated with differential DNAm in genes involved in metabolism, inflammation, immune response, and cardiac development [13]. Although no individual CpG sites reached genome-wide significance, these findings suggest that *in utero* environmental exposures may influence congenital heart defect risk through epigenomic pathways, underscoring the need for more mechanistic studies to establish mediating links.

Interestingly, data from the BEAR study demonstrated that iAs exposure was associated with altered DNAm of imprinted genes, including potassium voltage-gated channel subfamily Q member 1 (*KCNQ1*) [8]. Because imprinted genes have only one active allele while the other is silenced, they are particularly vulnerable to epigenetic disruption. Due to the timing of epigenetic marks on imprinted gene regions (e.g. prior to zygotic formation), these results from the human study (BEAR cohort) guided the design of complementary mouse studies in which animals were exposed to iAs preconceptionally. Supporting the hypothesis, preconception iAs exposure in mice resulted in altered DNAm of imprinted genes, including *Kcnq1*, and genes implicated in diabetes development [38, 39]. Moreover, these findings revealed that both parental and grandparental exposures produced persistent DNAm alterations in these loci. Notably, the results from the human population study influenced the design and testing in murine toxicologic experiments.

Importantly, *KCNQ1*/*Kcnq1* has emerged as a consistently affected locus across species. In humans, altered methylation of *KCNQ1* has been linked to metabolic disorders, including type 2 diabetes and growth abnormalities [43]. In mice, *Kcnq1* disruption has been associated with impaired glucose tolerance and insulin signaling [38, 39, 44]. These cross-species findings underscore the conserved nature of *KCNQ1*’s susceptibility to epigenetic perturbation and highlight its potential role as a key mediator of transgenerational effects following toxicant exposures.

Together, these findings illustrate how maternal exposures, inflammation, and sex-specific biology converge on the epigenome to shape neonatal health outcomes. Identifying and validating epigenetic biomarkers for conditions such as PTB, ROP, CLD, and TOF holds promise for advancing early risk assessment and guiding targeted interventions to improve infant health.

## Child health outcomes

While neonatal health outcomes represent the earliest consequences of environmental exposures, the influence of the epigenome extends well beyond the perinatal period into childhood. Epigenetic modifications established *in utero* or during early life can persist, shaping developmental trajectories and disease susceptibility across multiple systems. Maternal exposures such as iAs and air pollution have been implicated in altering DNAm and miRNAs that regulate key pathways in neurodevelopment and cardiac health [7, 13]. Additionally, epigenetic alterations have been associated with a wide range of child health outcomes including increased risk of neurodevelopmental disorders [e.g. impaired cognition, autism spectrum disorder (ASD) and attention-deficit/hyperactivity disorder (ADHD)], obesity, and heightened susceptibility to asthma [7, 45–51]. Together, these findings highlight the pivotal role of the epigenome in mediating how early environmental insults shape child health outcomes. Table 3 summarizes the selected scientific findings describing how epigenetic modifications can shape child health trajectories.

**Table 3:** Epigenetic studies focusing on child health outcomes.^a^

Study	Exposure or condition	Biological sample	Epigenetic changes	Health or developmental outcome	Sex-specific	Cohort
Tilley *et al*. [45]; PMCID: 5841757	Epigenetic signature	Placental tissue	Altered DNAm	Placental DNAm signature predicts cognitive function at age 10		ELGAN
Meakin *et al*. [46]; PMCID: 6354776	Epigenetic signature	Placental tissue	Altered DNAm	DNAm altered in placental HPA-axis genes related with cognitive impairment at age 10		ELGAN
Freedman *et al*. [47]; PMCID: 9475498	Epigenetic signature	Placental tissue	Altered miRNA	Placental miRNAs used to predict cognitive impairment at age 10		ELGAN
Santos *et al*. [48]; PMCID: 7730750	Multi-omics predictors	Placental tissue	Altered DNAm and miRNA	Predicted social and cognitive function: autism risk, IQ, social impairments		ELGAN
Wilmot *et al*. [49]; PMCID: 4724325	ADHD diagnosis	Saliva	Altered DNAm	Epigenetic biomarkers of ADHD	Yes	Boys age 7–12
Clark *et al*. [50]; PMCID: 6773381	Epigenetic signature	Placental tissue	Altered DNAm	Childhood obesity risk	Yes	ELGAN
Li *et al*. [51]; PMCID: 12106279	Epigenetic signature	Newborn blood	Altered DNAm	Childhood asthma risk	Yes	ECHO
Martin *et al*. [52]; PMCID: 5331919	Sex-specific DNA methylation	Placental tissue	Altered DNAm	Sex-dependent epigenetic patterning in the placenta related to immune function	Yes	ELGAN + NHBC
Beck *et al*. [7]; PMCID: 7036137	Inorganic arsenic	Plasma	Altered DNAm	Cardiovascular disease and diabetes		Mexico (Zimapán, Lagunera)

a Summary of selected studies linking maternal exposures during pregnancy to child health outcomes through epigenetic mechanisms. Columns indicate the exposure or condition assessed, biological sample analyzed, observed epigenetic change, associated health or developmental outcome, evidence of sex-specific effects, and the study cohort. Epigenomic alterations include altered DNA methylation (DNAm) and microRNA (miRNA). ADHD: attention-deficit/hyperactivity disorder; iAs: inorganic arsenic; IQ: intelligence quotient; HPA: hypothalamic-pituitary-adrenal; ELGAN: Extremely Low Gestational Age Newborn cohort; ECHO: Environmental influences on Child Health Outcomes cohort; NHBC: New Hampshire Birth Cohort; NC Birth Defect: North Carolina Birth Defects cohort.

Epigenetic regulation in the placenta has been associated with cognitive and neurodevelopmental outcomes. In the ELGAN cohort, distinctive placental hypermethylation patterns at specific CpG sites, including those within neurodevelopmental genes, were shown to predict cognitive performance at age 10 [45]. A follow-up analysis from the same cohort similarly found that differential DNAm in 10 genes related to the hypothalamic-pituitary-adrenal (HPA) axis was associated with cognitive function at age 10 [46]. These findings suggest that placental epigenetic programming may have long-term consequences for cognitive trajectories.

Epigenetic contributions to cognition extend beyond DNAm. In the ELGAN cohort, placental miRNA profiles, particularly those that regulate genes involved in inflammation and apoptosis, were associated with cognitive performance at age 10 among extremely preterm children [47]. Integrative multi-omics analyses from the same cohort, combining placental mRNA, miRNA, and DNAm data, demonstrated even greater predictive capacity, linking placental biology to both intellectual and social outcomes and implicating epigenetic programming in ASD risk [48]. Beyond ASD, altered DNAm in genes involved in inflammation and neurotransmission pathways has been reported in boys aged 7–12 years with attention-deficit/hyperactivity disorder (ADHD), suggesting shared molecular mechanisms across neurodevelopmental disorders [49]. Collectively, these findings underscore the placenta–brain axis as a central mechanism within the DOHaD framework.

Epigenetic programming in the placenta has also been associated with metabolic outcomes. In the ELGAN cohort, changes in placental DNAm were associated with childhood BMI trajectories, suggesting that these marks may predict obesity risk established *in utero* [50]. Such findings highlight the role of the placenta in long-term growth and energy regulation and point to placental methylation signatures as potential early biomarkers of obesity and related metabolic dysfunction.

Similarly, the epigenome plays a central role in immune regulation and respiratory health. In the ECHO cohort, DNAm patterns in newborn blood were associated with later asthma risk, particularly among extremely preterm infants [51]. These associations highlight how early-life epigenetic variation can predispose children to immune dysregulation and inflammatory airway disease. Given the established links between maternal environmental exposures, altered epigenetic programming, and immune system pathways, DNAm signatures hold promise as early indicators of asthma susceptibility and targets for preventive interventions.

Together, these findings demonstrate that epigenetic programming serves as a lasting molecular imprint of environmental exposures, with consequences extending from neurodevelopment to metabolism, and immunity. Many of these epigenetic changes are sex-specific. For example, sex-dependent placental patterning has been linked to immune function in placental samples from the ELGAN and New Hampshire Birth Cohort (NHBC) [52]. The identification of DNAm and miRNA biomarkers for outcomes such as cognition, ASD, ADHD, obesity, and asthma underscores the potential of the epigenome as both a mechanistic link and a predictive tool for child health outcomes. Supporting this concept, an independent study of placental samples from a cohort of children in Mexico reported that *in utero* iAs exposure was linked to altered DNAm in cardiovascular disease and diabetes risk, further reinforcing the potential of methylation patterns at key loci to serve as predictive biomarkers of later health outcomes [7].

## Summary and future directions

### Current insights

Environmental epigenetics is a transformative framework for understanding how maternal exposures shape pregnancy, neonatal, and child health outcomes. A growing body of evidence demonstrates that early-life exposures, including metals, air pollution, microorganisms, and socioeconomic stressors, leave measurable and biologically meaningful imprints on the placental and fetal epigenome (Table 4). These epigenetic modifications regulate gene expression and contribute to outcomes such as preeclampsia, PTB, neurodevelopmental disorders, asthma, and altered metabolic and immune function. Importantly, the identification of sex-specific epigenetic responses highlights the biological nuance of developmental susceptibility and positions the placenta as a central mediator of exposure-related risk. Collectively, these insights underscore the potential of placental and fetal epigenetic signatures as biomarkers for predicting long-term health trajectories and as targets for preventive strategies. In this invited perspective, studies from our research group are emphasized; still the findings are consistent with the broader body of evidence summarized in recent reviews, underscoring the predictive relevance of placental and neonatal epigenetic markers for childhood outcomes [17–19]. Building on this, reviews of the placental epigenome within the DOHaD framework highlights that large, multi–cohort studies across the USA and globally have demonstrated reproducible associations between prenatal exposures (including metals, air pollution, and maternal stress) and placental DNAm. Importantly, they show that these epigenetic signatures predict child outcomes such as neurodevelopment, metabolic regulation, and immune function, underscoring that the sex–specific and exposure–related patterns observed in our cohorts are echoed across diverse populations [19].

**Table 4:** Summary of exposures and conditions across developmental stages associated with epigenetic modifications and health outcomes.^a^

Outcomes	Exposure or condition	Biological sample	Epigenetic changes	Health or developmental outcomes
Pregnancy-related outcomes	Metals (Cu, Mn, iAs, Cd) Pharmaceuticals Placental microbiome Socioeconomic adversity Maternal BMI Preeclampsia Gestational diabetes	Placental tissue Umbilical cord tissue Umbilical cord blood	DNAm alterations miRNA dysregulation Altered epigenetic clock	Placental nutrient and chemical transport Placental cell growth Placental angiogenesis Placental function Immune regulation Inflammation pathways Preeclampsia
Neonatal health outcomes	Metals (Cd, Fe, Zn, iAs) Preterm birth Maternal age Systemic inflammation Retinopathy of prematurity Chronic lung disease Gestational age and sex	Placental tissue Umbilical cord blood Maternal blood Neonatal blood	DNAm alterations miRNA dysregulation Altered epigenetic clock	Lower birth weight Gestational age Smaller head circumference Cancer, diabetes, and immune function Preterm birth Inflammation Retinopathy of prematurity Chronic lung disease Tetralogy of Fallot
Childhood health outcomes	Epigenetic signatures ADHD Air pollution Metals (iAs)	Placental tissue Saliva Newborn blood Newborn dried blood spots	DNAm alterations miRNA dysregulation	Reduced cognition Autism spectrum disorder ADHD Obesity Asthma Cardiovascular disease Diabetes

a Epigenomic alterations include altered DNA methylation (DNAm), microRNA (miRNA), and epigenetic clocks. iAs: inorganic arsenic; Cu: copper; Mn: manganese; Cd: cadmium; Fe: iron; Zn: zinc; BMI: body mass index; ADHD: attention-deficit/hyperactivity disorder.

### Scientific challenges

Despite the considerable scientific progress summarized in this perspective, several challenges continue to hinder advancement in understanding how early–life environments shape long–term health. Across epidemiologic studies, variability in exposure measurement, differences in analyzed tissue types, and inconsistent outcome definitions limit meaningful cross–cohort comparisons and weaken the strength of resulting conclusions. Moreover, few longitudinal cohorts have the depth and duration of follow–up needed to evaluate the persistence and functional relevance of epigenetic marks across the life course.

The ELGAN cohort is not immune to limitations—its population consists exclusively of children born extremely preterm, and findings may not fully generalize to term cohorts. As in all observational studies, uncontrolled confounding remains a concern, and observed epigenetic differences may reflect correlated biological responses rather than causal mediators. Additional methodological challenges, including selection bias, collider bias, fixed–cohort bias, and other structural sources of error, further complicate interpretation [53]. Cross–sectional comparisons of placentas from preterm and term births add yet another layer of complexity, as epigenetic variation may reflect gestational age rather than underlying pathology.

Yet ELGAN directly addresses many of the field’s most persistent limitations. Its harmonized biospecimen collection protocols reduce heterogeneity in exposure and tissue assessment. Its deeply phenotyped cohort and standardized outcome definitions enhance internal consistency and improve interpretability. Most importantly, ELGAN’s extensive longitudinal follow–up—from birth through childhood and adolescence—provides a rare opportunity to evaluate the durability and functional relevance of early–life epigenetic marks, something few cohorts can achieve. While replication resources remain limited, ELGAN’s design uniquely enables the integration of placental biology with later neurodevelopmental, metabolic, and immune outcomes, offering insights that would be difficult to obtain elsewhere.

### Critical scientific advances

Recent breakthroughs in environmental epigenetics have introduced powerful tools and assessments that are reshaping our understanding of early-life exposures. Epigenetic clocks, such as eGA and AgeAccelGrim, have emerged as promising biomarkers of biological aging, with predictive value for outcomes including PTB and adolescent blood pressure [15, 21, 32, 48].

In parallel, multi-omic approaches that integrate DNAm, miRNA, mRNA, protein, and metabolite data have significantly enhanced our ability to predict neurodevelopmental outcomes, such as cognitive impairment, ASD, and ADHD [45–49]. These integrative analyses represent a major step forward in capturing the systems-level effects of environmental exposures during critical developmental windows.

**Figure 3:**
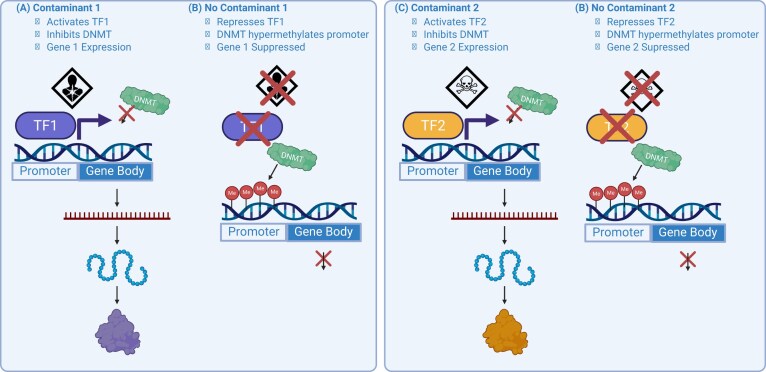
The transcription factor occupancy theory for gene-specific CpG methylation. Illustration of the transcription factor occupancy theory, which posits that environmental contaminants influence gene-specific patterns of CpG methylation and subsequent mRNA expression by modulating transcription factor (TF) activity. (A) Exposure to Contaminant 1 activates transcription factor TF1, which binds to the promoter region of Gene 1. TF1 occupancy inhibits DNA methyltransferase (DNMT) activity, preventing promoter hypermethylation and enabling transcription. (B) In the absence of Contaminant 1, the TF1 remains inactive and does not bind to the promoter. DNMT is able to hypermethylate the promoter region, resulting in gene repression. (C) Exposure to Contaminant 2 activates transcription factor TF2, which binds to the promoter region of Gene 2. TF2 occupancy inhibits DNMT activity, allowing for gene expression. (D) In the absence of Contaminant 2, the TF2 is repressed and does not occupy the promoter. DNMT is able to hypermethylate the promoter region, leading to gene repression.

**Figure 4:**
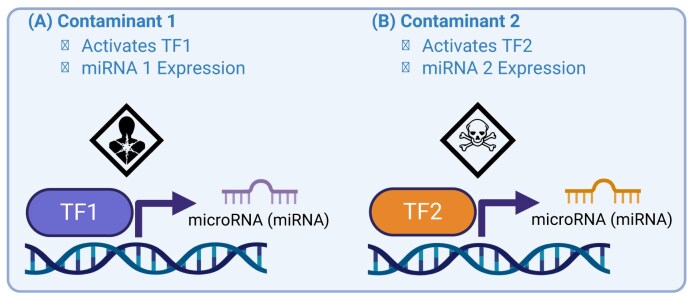
Transcription factor activation and microRNA expression. Conceptual illustration of how environmental factors modulate transcription factor (TF) activity, downstream microRNA (miRNA) expression, and subsequent gene expression. (A) Contaminant 1 activates transcription factor TF1, which binds to regulatory regions and induces expression of microRNA 1. (B) Exposure to Contaminant 2 activates transcription factor TF2, leading to induction of microRNA 2.

Importantly, this perspective highlights studies demonstrating that epigenetic modifications, particularly altered CpG methylation and miRNA expression, are not random in their location in the genome. Instead, these modifications are enriched in functionally relevant regions, including loci containing specific TF binding sites. Among the studies to identify that chemical exposures are associated with gene-specific DNAm patterns, or “footprints,” is Sanders *et al*. [24], which identified conserved DNA motifs within genes exhibiting cadmium-associated DNAm changes. These exposure-associated epigenetic “footprints” are consistent with the TF occupancy theory. To elaborate, patterns of CpG methylation and miRNA expression likely reflect underlying TF binding events [54–59]. According to the TF occupancy model, TF binding can locally influence the accessibility or activity of epigenetic machinery or directly induce miRNA transcription, thereby providing a mechanistic link between environmental exposures and site-specific epigenetic regulation [60]. Supporting this framework, polycyclic aromatic hydrocarbons activate the aryl hydrocarbon receptor (AhR), leading to altered DNAm at target genes and corresponding changes in gene expression [57]. Oxidative stress activates nuclear factor erythroid-derived 2–like 2 (NRF2), which modulates the expression of epigenetic regulatory enzymes, including DNA methyltransferases (DNMTs) and histone deacetylases, thereby linking antioxidant responses to changes in DNAm and gene expression [58]. Hypoxic conditions activate hypoxia-inducible factor 1 (HIF-1), influencing the expression of miRNAs that regulate angiogenesis and metabolism [59]. Collectively, these examples demonstrate that TFs can influence CpG methylation patterns and miRNA expression at specific genomic loci, illustrating both shared and exposure-specific epigenetic responses. Figure 3 illustrates how environmental exposures can regulate gene expression through TF-mediated inhibition of DNAm, highlighting a potential epigenetic mechanism by which environmental signals influence transcriptional activity. Figure 4 presents a complementary mechanism, demonstrating how environmental signals may affect post-transcriptional gene regulation via TF-mediated control of miRNA expression. These mechanistic pathways underscore how the epigenome mediates environmental effects on maternal and child health. The targeted nature of these interactions highlights the importance of incorporating TF analysis into epigenetic studies to better understand the biological consequences of early-life exposures.

### Key unanswered questions

Several critical questions remain at the forefront of environmental epigenetics. Which epigenetic changes are mediators versus correlative? Can we identify modifiable marks that respond to interventions? How do sex-specific epigenetic responses influence disease susceptibility across the lifespan? Addressing these questions will require longitudinal studies with deep phenotyping, mechanistic validation, and the inclusion of diverse populations to ensure generalizability of findings.

### Future directions

Looking ahead, several opportunities will shape the next phase of discovery. Longitudinal birth cohorts with extended follow-up into adolescence and adulthood are essential to assess whether placental and fetal epigenetic signatures at birth reliably predict neurodevelopmental, metabolic, and immune outcomes. Additionally, multi-omic integration will be critical for identifying molecular pathways that link maternal exposures to child health trajectories, while mechanistic studies will clarify mediating relationships and identify targets for intervention.

### Clinical translation and impact

Ultimately, the future of environmental epigenetics lies in its integration into predictive and preventive medicine. By positioning the epigenome at the center of exposure-related risk, researchers can develop biomarkers for the early identification of at-risk children, guide targeted interventions, and potentially explore epigenetic reprogramming as a therapeutic strategy. Such approaches may help reverse or mitigate exposure-induced molecular changes, transforming our understanding of the developmental origins of health and disease. In doing so, environmental epigenetics has the potential to inform actionable strategies that improve maternal and child health across generations.
